# Stress and social support systems among final year medical students of Medical University of Silesia

**DOI:** 10.1186/1753-6561-9-S1-A57

**Published:** 2015-01-14

**Authors:** EO Fatoba, D Bzdzikot

**Affiliations:** 1 Department of Psychiatry, Medical University of Silesia, Katowice, Poland; 2 Medical University of Silesia, Department of Psychiatry, 18 Medyków Street, 40-752 Katowice, Poland

## Background

Medical education is tedious and takes both a physical and psychological toll on medical students. Stress could lead to burnout which research has shown that is prevalent among medical students and can lead to other significant dangers if it continues into residency and beyond [1]. Aims: Among potential interventions to prevent stress and its harmful effects among medical students, a good social support system is essential. Our aim is to assess support systems among different demographics of final year medical students of SUM.

## Methods

Interviews and a self-administered anonymous survey of 55 final year medical students by a 2 part questionnaire assessing demographics and support system were carried out.

## Results

35 final year medical students responded equating to a response rate of 76%. 54% were male mirroring class demographics. The majority (69%) were more than 25 years old and unmarried (89%). We observed that majority of students relied on family, friends and classmates for support when stressed and very few relied on mentors, faculty and school administration for support.

## Conclusions

The major support systems relied upon by the students has been identified and a void of support from mentors, faculty and school administration discovered. Further research for the cause of this might be helpful to distinguish between the lack of support provision or lack of use on the part of students. This is important as healthy support systems are necessary to cope with the stress ahead in the field of medical practice.
